# Fluid Mechanics in Dentinal Microtubules Provides Mechanistic Insights into the Difference between Hot and Cold Dental Pain

**DOI:** 10.1371/journal.pone.0018068

**Published:** 2011-03-23

**Authors:** Min Lin, Zheng Yuan Luo, Bo Feng Bai, Feng Xu, Tian Jian Lu

**Affiliations:** 1 School of Aerospace, Biomedical Engineering and Biomechanics Center, Xi'an Jiaotong University, Xi'an, People's Republic of China; 2 State Key Laboratory of Multiphase Flow in Power Engineering, Xi'an Jiaotong University, Xi'an, People's Republic of China; 3 Department of Medicine, HST-Center for Biomedical Engineering, Brigham and Women's Hospital, Harvard Medical School, Boston, Massachusetts, United States of America; University of Oxford, United Kingdom

## Abstract

Dental thermal pain is a significant health problem in daily life and dentistry. There is a long-standing question regarding the phenomenon that cold stimulation evokes sharper and more shooting pain sensations than hot stimulation. This phenomenon, however, outlives the well-known hydrodynamic theory used to explain dental thermal pain mechanism. Here, we present a mathematical model based on the hypothesis that hot or cold stimulation-induced different directions of dentinal fluid flow and the corresponding odontoblast movements in dentinal microtubules contribute to different dental pain responses. We coupled a computational fluid dynamics model, describing the fluid mechanics in dentinal microtubules, with a modified Hodgkin-Huxley model, describing the discharge behavior of intradental neuron. The simulated results agreed well with existing experimental measurements. We thence demonstrated theoretically that intradental mechano-sensitive nociceptors are not “equally sensitive” to inward (into the pulp) and outward (away from the pulp) fluid flows, providing mechanistic insights into the difference between hot and cold dental pain. The model developed here could enable better diagnosis in endodontics which requires an understanding of pulpal histology, neurology and physiology, as well as their dynamic response to the thermal stimulation used in dental practices.

## Introduction

Dental pain is a significant health problem which negatively affects the lives of millions of people worldwide and induces huge societal costs [1]. Although dental thermal pain has become an increasingly mature topic of study and the sensory responses of tooth to various stimulations have been studied for decades [2]-[7], frustration is mounting over the limited breakthroughs in dental pain therapy, mainly due to the limited acknowledge of dental pain mechanism [8].

**Figure 1:**
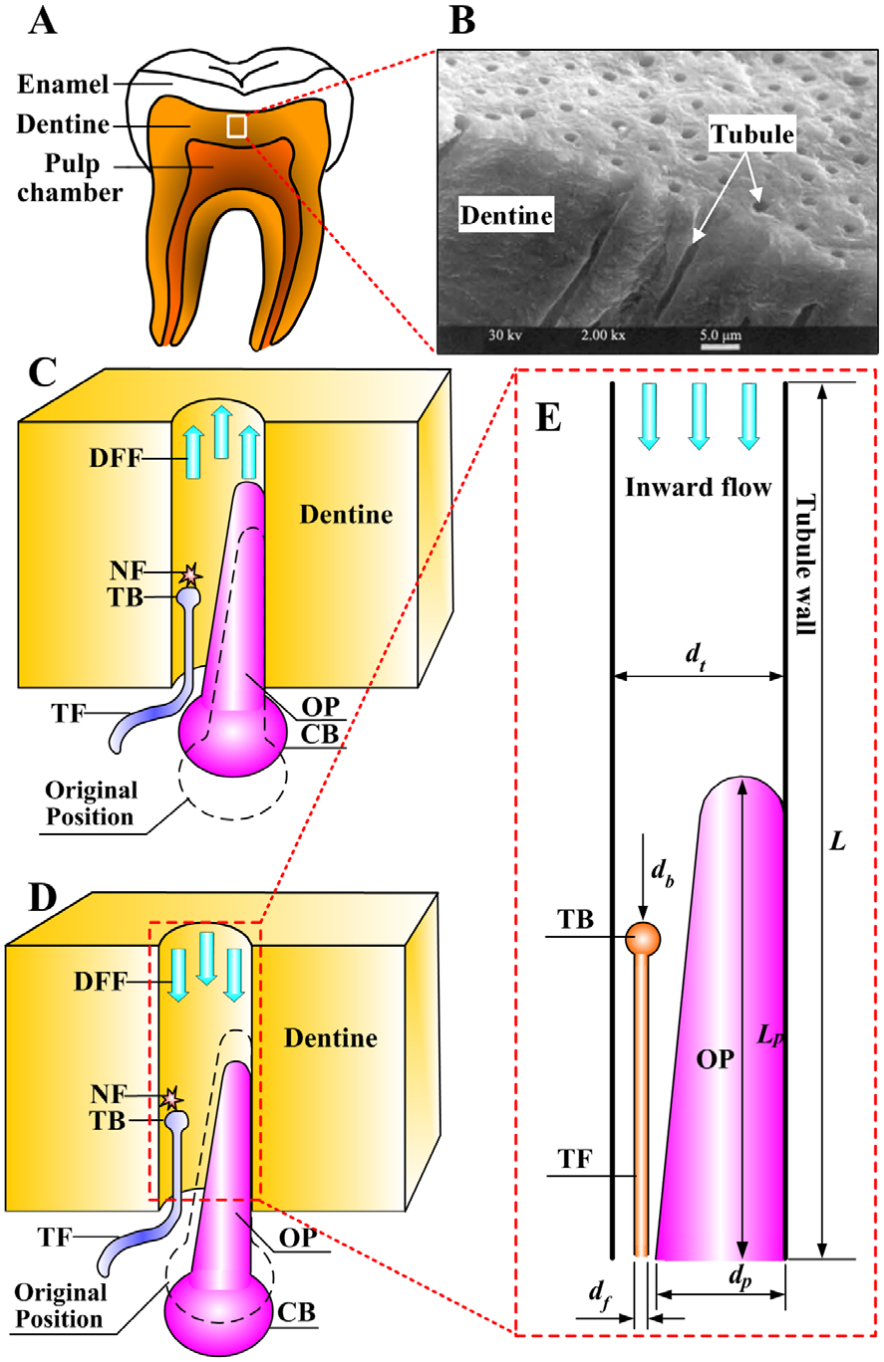
Physiological relevant structures. (*A*) Cut-away image of human tooth; (*B*) SEM image of dentine showing solid dentine material and dentinal microtubules (DMTs) running perpendicularly from pulpal wall toward dentine-enamel junction [9]. (*C*) Schematic of DMT innervation system and nerve firing (NF) in response to outward dentinal fluid flow (DFF). Terminal fibril (TF) situated in tubule between odontoblast process and tubule wall [11]. Slightly outward displacement of odontoblastic process (OP) and its cell body (CB) in response to outward flow. The dash line indicates the original position of the odontoblast. The outward movement of the OP reduces the dimension of the channel available for the DFF, resulting in increased shear stress on the terminal bead (TB) although the volume flow is low [4]. (*D*) Slightly inward displacement of OP in response to inward flow. This movement tends to produce a smaller shear stress on the TB than that at its original position (dash line). (*E*) Physically realistic model for fluid dynamics simulation (inward flow). *d*_t_, *d*_p_ and *d*_f_ are diameters of DMT, OP and TF, respectively; *R*_b_ is radius of TB; *L* is computational length. One side of OP surface is in contact with tubular surface [36], hence no dentinal fluid is allowed to pass through at this side. The TF and OP are modeled as rigid structures that do not deform due to DFF. We assumed that there is no synaptic structure between OP and TF [48], though different finding has been reported [36]. TB containing varying amounts of receptor organelles [35] is assumed as the sensory zone at the end of TF. The volume of TF is smaller as compared with odontoblast [36], and hence the movements of TF as caused by DFF is neglectable.

Studies of tooth microstructure have revealed that dentinal microtubules radiate from pulp wall to exterior cementum or dentine-enamel junction (DEJ) (Fig. 1 *A* and *B*) [9]. Most of the dentinal microtubules contain non-myelinated terminal fibrils and odontoblastic processes (extension of odontoblast) that are placed in an environment filled with dentinal fluid [4], [10], [11]. Based on the characteristics of tooth innervation system, three main theories have been proposed to explain the mechanisms underlying dental pain sensation [12]: (i) Neural theory, which assumes that changes in tooth surface temperature are conducted through enamel, dentin and finally to sensory receptors located at DEJ causing neuron excitation; (ii) Odontoblastic transduction theory, which assumes external stimulus is transmitted along odontoblasts and transferred to nerves via synaptic junctions between odontoblasts and nerves; (iii) Hydrodynamic theory, which attributes dental pain sensation to the stimulation of mechano-sensitive nociceptors as a consequence of dentinal fluid movement within dentinal microtubules. Among these hypotheses, the hydrodynamic theory is the most widely accepted explanation for dental pain sensation [3], [4], [7].

External stimuli (*e.g.*, thermal, mechanical and dental restorative processes) applied to human tooth cause either an inward (toward the pulp chamber) or outward (away from the pulp chamber) dentinal fluid flow in dentinal microtubules [13]–[16]. Dentinal fluid flow-induced shear stress on intradental nerve terminals may activate mechano-sensitive ion channels (*e.g.*, ASIC3, TREK1 and TREK2) [17] and cause dental pain sensation [17]–[20]. Direct evidence for the hydrodynamic theory is that intradental neural discharge rate increases with increasing dentinal fluid flow velocity (cat tooth, *in vivo*) [4], [7]. However, the neural responses in tooth (human and cats, *in vivo*) have been found to be more sensitive to the outward fluid flow than to the inward fluid flow [3], [4], [7] and that cold stimulation evokes more rapid transient pain sensations whilst hot stimulation generally induces a dull lasting pain [21], [22], both outlive the hydrodynamic theory. Although the precise transduction mechanism remains unknown, the major differences between the effects of hot and cold stimulations have been identified: the former causes an inward fluid flow while the latter causes an outward fluid flow [4], [7], [16]. An initially high rate of outward fluid flow under cooling was found to correspond to short latency neural responses [23]. In addition, odontoblastic process movements as aspirated by dentinal fluid flow have been demonstrated [4], [24]. Hence, a better understanding of the fluid flow, odontoblastic process movement and the associated intradental nerve responses would provide an insight into the mechanisms underlying the different responses of tooth to cold and hot stimulations.

In the present study, the effects of dentinal fluid flow on the shear stress experienced by nerve terminals were firstly analyzed using a computational fluid dynamics (CFD) model. A modified Hodgkin-Huxley (H-H) model was then proposed to simulate the intrapulpal nociceptor transduction. We validated the developed models by comparing the simulated results with the experimental observations by Andrew & Matthews [4] and Vongsavan & Matthews [7]. Based on the simulated results, we explained in detail that dentinal fluid flow with different directions and odontoblastic process movements cause significantly different intradental neural responses. Finally, mechanistic insights into the difference between hot and cold dental pain responses were provided.

## Results and Discussion

**Figure 2:**
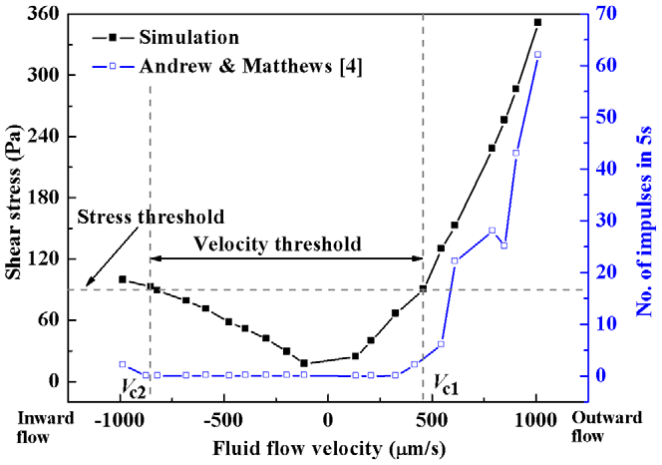
Variation of TB MSS (simulated) and neural discharge rate (measured[**4**]) as a function of fluid velocity (negative for inward flow; positive for outward flow). Velocity thresholds: *V*_c1_ = 460.4 µm/s, *V*_c2_ = −849.9 µm/s [4].

The tooth is innervated almost exclusively by nociceptive afferents [25], [26]. The neural discharge thresholds expressed in terms of flow velocity in a single dentinal microtubule have been found to be 460.4 µm/s (in the case of outward flow) and −849.9 µm/s (in the case of inward flow) [4]. However, there exists no experimental method for determining the mean mechanical pain threshold of intradental nociceptors. Since the mechano-sensitive ion channels open at a specific mechanical threshold [27], the two critical flow velocities should generate the same maximum shear stress on the terminal bead (*τ*_MSS_), which was assumed to be the threshold shear stress, *τ*_thr_, in this study. Therefore, our strategy was to relate the two critical flow velocities with *τ*_thr_. The local channel diameter (distance between terminal bead and odontoblastic process) depends upon the odontoblastic process displacement, which, in turn, depends upon the velocity and direction of the fluid flow. For example, when an inward flow is applied, a higher rate of fluid flow will cause more significant odontoblastic process displacement, increasing thereby the dimension of the channel available for the fluid flow [18] (Fig. 1 *D*). The above-mentioned relationships were simplified and simulated as follows. One value was specified for the local channel diameter at the critical outward flow velocity of 460.4 µm/s and the corresponding *τ*_MSS_ (predicted) was assumed to be *τ*_thr_. Then, the value of the local channel diameter at the critical inward velocity of −849.9 µm/s was adjusted over several cycles of *τ*_MSS_ predictions until the predicted *τ*_MSS_ was the same as the one obtained at the flow velocity of 460.4 µm/s. The local channel diameter values corresponding to other flow velocities (except the two critical flow velocities) were obtained with linear interpolation. The predicted *τ*_MSS_ as a function of flow velocities are shown in Fig. 2. It should be noted that the *τ*_thr_ and the predicted *τ*_MSS_ at other velocities vary with the specified local channel diameter value at the flow velocity of 460.4 µm/s (Figure S1). Nevertheless, such variation does not affect the predicted neural responses (Figure S2, Text S1).

To qualitatively explain why the inward and outward flows evoke significantly different intradental neural responses, we plotted the *τ*_MSS_ against the flow velocities, and the results are compared with the change of neural responses (Fig. 2). We observed that the *τ*_MSS_ in the outward flow case increases dramatically with increasing flow velocity, corresponding to the increase in neural discharge rate. In sharp contrast, the *τ*_MSS_ in the case of inward flow was observed to be “inert” to the increasing flow velocity and the neural discharge rate is zero or low accordingly (Fig. 2). The results of Fig. 2 indicate that the distinct difference in intradental neural responses to different fluid flow directions may be attributed to odontoblastic process displacement. *τ*_MSS_ is dependent upon the fluid velocity, which, in turn, is dependent upon the dimension of the channel available for the fluid flow. Reducing the channel diameter may result in a higher shear stress and thus the neural discharge rate, although the volume flow is low.

**Figure 3:**
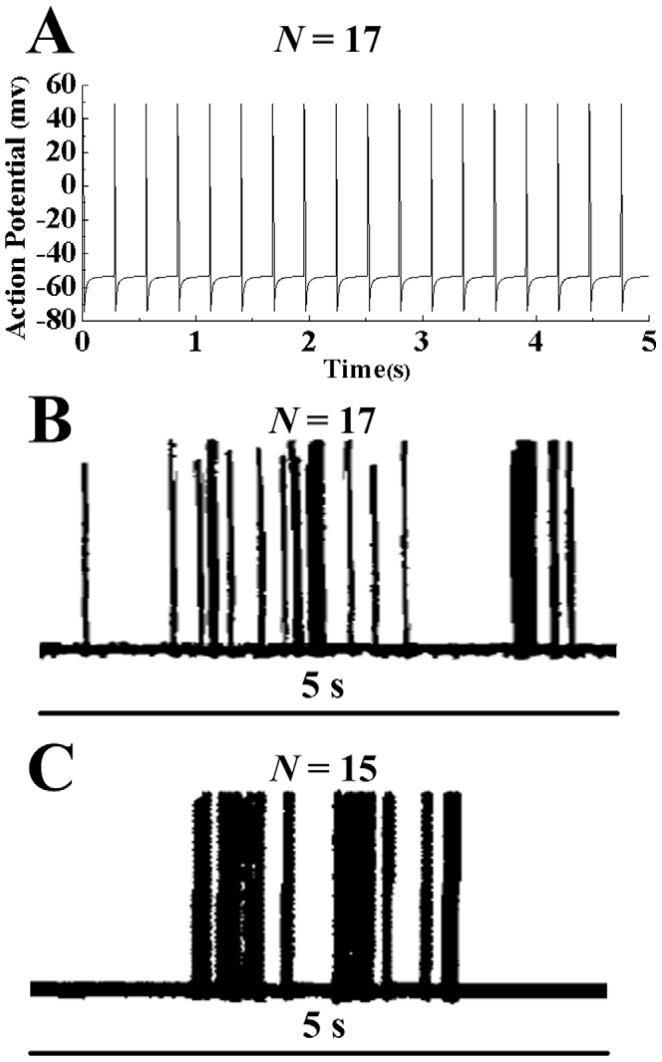
Response of nociceptor membrane potential in cat tooth to flow velocity of ∼611.6 µm/s. (*A*) Action potential simulated with the modified H-H model. (*B* and *C*) Experimental measurements by Vongsavan & Matthews [7] and Andrew & Matthews [4], respectively. *N* is the number of neural impulses in 5 s.

The simulated results of the membrane potential and frequency response at the outward flow velocity of ∼611.6 µm/s are shown in Fig. 3 *A*. The simulated impulse frequency (*N* = 17) agrees well with the experimental measurements of Vongsavan & Matthews [7] (*N* = 17 in Fig. 3 *B*) and Andrew & Matthews [4] (*N* = 15 in Fig. 3 *C*). It is known that the pain intensity is reflected by the frequency of the impulse, not by its exact magnitude or shape. Hence, the present model is capable of capturing the neural responses of intradental mechano-sensitive nociceptors.

**Figure 4:**
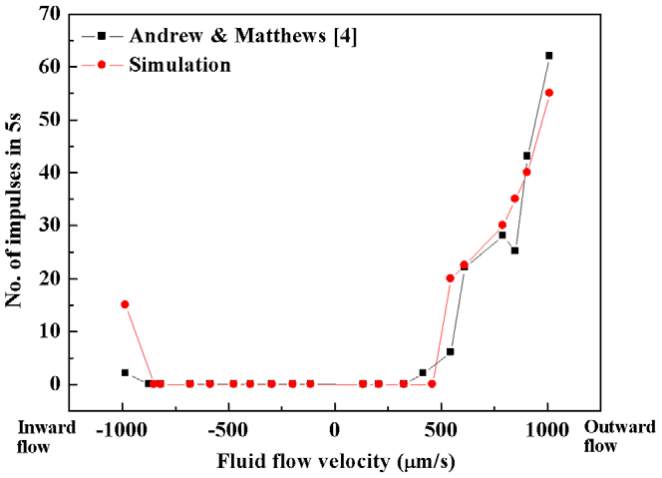
Comparison of frequency response between experimental measurements[**4**] and model predictions. Note that cold stimulation (0∼5°C) is reported to cause outward flow velocities range between 531.2∼849.9 µm/s [2], [4], whilst hot stimulation (∼55°C) causes inward flow velocities range between 354.1∼779.1 µm/s [4].

To quantitatively explain the significant difference in intradental neural responses to different fluid flow directions, we modeled the neural discharge rate (in 5 s) under different fluid flow velocities. The results are shown in Fig. 4. Experimental observations show that nociceptors respond in a significantly different manner to dentinal fluid flow having different directions [4]. The neural discharge rate increases progressively as the outward flow velocity increases above the threshold. In contrast, the nociceptors show much less sensitivity to the inward flow. The simulated results are in good agreement with the experimental data. Our simulations reveal that the odontoblastic process displacement accounts for the difference in the response of intradental nociceptors to inward and outward flows. The outward flow tends to carry the odontoblastic process toward the dentinal microtubule, reducing the dimension of the space for the fluid flow (Fig. 1 *C*) and thereby, increasing the fluid velocity (around the terminal bead wall) and the *τ*_MSS_. In this case, the neural discharge rate will increase even though the fluid velocity is low at the boundary. Conversely, the odontoblastic process displacement in the inward flow case tends to increase the dimension of the space for the fluid flow around the terminal bead wall (Fig. 1 *D*), resulting in a lower *τ*_MSS_ even though the fluid velocity at the boundary is relatively high. Therefore, the intradental nociceptors exhibit “low sensitivity” to the inward flow.

Although thermal pain sensation has been attributed to the activation of thermal-gated ion channels [28], it may not be exactly the case when dental pain evoked by thermal stimulation is considered [23]. It has been demonstrated that the sensory response of tooth to thermal agitation occurs before a temperature change can be detected in the pulp-dentine junction where most sensory structures are located [29]. If the hydrodynamic theory is a valid mechanism responsible for dental thermal pain, the difference in the subjective pain responses may result from the different dentinal fluid flow induced by cold and hot stimulations. Although fluid velocities were employed as the boundary conditions in the modeling of the *τ*_MSS_ and the subsequent neural discharge, the difference between hot and clod dental pain sensations can still be revealed. Note that the cold stimulation (0∼5°C)-induced outward flow velocities range between 531.2∼849.9 µm/s [2], [4] whilst the velocity range of the inward flow becomes 354.1∼779.1 µm/s [4] in the case of hot stimulation (∼55°C). These experimentally reported inward and outward flow directions and their corresponding magnitudes were consistent with those employed as the boundary conditions in this study (Fig. 2 and 4). Based on the simulated results (Fig. 4), the explanations for the phenomenon that cold stimulation causes sharper and more shooting pain sensation than does hot stimulation may be summarized as follows.

The intradental neural response was observed after a short latency (<1 s) of cold stimulation (0∼5°C) [30], [31]. At this stage, the local temperature (where terminal bead is located) is still far from being capable of activating the thermo-sensitive nociceptors [31]. Therefore, it appears unlikely that such response is originated from the thermo-sensitive nociceptors. Note that fluid flow could be detected before a noticeable temperature change could be found in DEJ and that the latency of the initiation of the dentinal fluid flow (<1 s) [4], [16] (induced by either hot or cold stimulation) corresponds to the latency of the neural response. In addition, the flow velocity induced by cold stimulation may easily exceed the threshold [2], [4], which may activate the mechano-sensitive nociceptors (Fig. 2 and 4). Therefore, the initial stage of cold-induced dental pain (sharp, shooting pain) may involve the activation of mechano-sensitive nociceptors by dentinal fluid flow. It should be mentioned that, after a long latency (∼30 s), the neural response (dull, burning pain) to cold stimulation may be attributed to the activation of thermo-sensitive nociceptors [22], [32]: by then the temperature around the nociceptors may have exceeded the threshold.

In the case of hot stimulation (55°C), a relatively long latency (>10 s) of the neural response was observed [30], [31]. During this stage, no neural discharge could be detected [4], [30], [31]. This does not contradict with the conclusion that the dentinal fluid flow may evoke the neural response, since hot stimulation can hardly initiate a high rate of the fluid flow [4] needed for the activation of mechano-sensitive nociceptors (Fig. 2 and 4). It is possible that after such a long latency, the temperature around the thermo-sensitive nociceptors reaches the threshold [31], triggering the nociceptors and causing pain sensation [30], [31].

In conclusion, we have developed a simulation framework coupling a CFD model with a modified H-H model for the quantification of dental pain sensation (in terms of neural discharge) evoked by dentinal fluid flow in dentinal microtubules. By attributing to different dentinal fluid flow directions and the corresponding odontoblast movements, it is demonstrated that the proposed models are capable of explaining the experimental observation that intradental mechano-sensitive nociceptors are not “equally sensitive” to inward and outward flows of dentinal fluid [4], [7]. The mechanism underlying the phenomenon that cold stimulation evokes sharper and more shooting pain sensation than hot stimulation also involves dentinal fluid flow and odontoblast movement. To our best knowledge, this study is the first attempt to the quantitatively interpret dental thermal pain responses in terms of fluid mechanics and will be potentially guide lines for tooth hypersensitivity treatment.

## Methods

### Modeling of shear stress

The dentinal microtubule innervation system is consisted of dentinal fluid, non-myelinated sensory nerve fibril and odontoblastic process, as shown schematically in Fig. 1 *C* and *D*. To compare the simulated results with existing experimental data, the diameter of the microtubule was selected as *d*_t_ ≈0.73 µm (cat canine) [4]. The dentinal fluid has a composition similar to that of cerebrospinal fluid, with viscosity *µ* ≈1.55×10^−3^ Pa·s and density *ρ* ≈1010 kg/m^3^ [33]. Up to 50% of the microtubules located in the pulpal horn are innervated [25] and, in most cases, each microtubule contains only one beaded terminal fibril [11]. The terminal fibril extends about 100 µm into the microtubule above the pupal wall [34]. The diameter of the terminal fibril is *d*_f_ ≈0.1 µm [34]. The terminal beads have varying amounts of receptor organelles and the diameter of the bead is *d*_b_ ≈0.2 µm [35]. The microtubule is also penetrated by odontoblastic process, whose cell body lies at the opening of the microtubule on the pulpal wall [4], and there is only one odontoblastic process inside a microtubule accompanied by only one terminal fibril in most cases [36]. The extension of the odontoblastic process was found to be restricted to the inner half of the microtubule (∼200 µm) [34]. The outline of the odontoblastic process is smooth and not beaded [11], with diameter decreasing along its longitudinal direction, *i.e.*, from pulpal wall to DEJ [36]. For simplicity, the odontoblastic process diameter was assumed to vary linearly with its longitudinal direction, given that its maximum *d*_op_ (at pulpal wall) is smaller than 1 µm [18]. Outward fluid flow causes slight movement of odontoblasts toward the microtubule whereas inward flow causes the odontoblasts to move in the opposite direction [4] (Fig. 1 *C* and *D*). The movement of the terminal fibril can be neglected due to its small volume as compared with that of odontoblast [18], [36].

The movement of the odontoblastic process changes the dimension of the space for the fluid flow, affecting thus the shear stress on the terminal bead [18] (Fig. 1 *C* and *D*). To model the effect of odontoblastic process movement upon the shear stress on the terminal bead, it was assumed here that the odontoblastic process displacement in the fluid flow direction changes linearly with the flow velocity. This assumption is deemed adequate for illustrating the overall behavior of the odontoblastic process in response to the dentinal fluid flow, because a higher rate of fluid flow will lead to a larger odontoblastic process displacement [4]. The CFD model was employed to simulate the *τ*_MSS_ which will most probably exceed the mechanical threshold of the nociceptors, *τ*_th_. For CFD simulation, a physically realistic model representing the inward flow of dentinal fluid is shown in Fig. 1 *E*. Based on the *in vivo* structure of the dentinal microtubules innervation system described above and the symmetrical structure of the terminal bead and odontoblastic process in the longitudinally sectioned plane (along their axes), the three-dimensional (3D) structure of fluid flow through the dentinal microtubule innervation system was simplified to a two-dimensional (2D) model. Since the focus of the present research was on the *τ*_MSS_, this simplification provides reasonable approximation for the numerical simulation of fluid dynamics. Steady state Navier-Stokes equations for incompressible and laminar flow were employed to model the shear stress experienced by the terminal bead, expressed as:
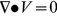
(1)

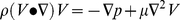
(2)where *V* (m/s), *p* (Pa), *ρ* (kg/m^3^) and *µ* (Pa·s) are the velocity vector, pressure, density and viscosity of dentinal fluid, respectively. Constant fluid velocity boundary conditions were applied in the simulation and the values of the fluid flow velocities employed were adopted from literature [4]. The experimentally recorded fluid flow velocities (nl s^−1^ mm^−2^) were converted into the fluid flow velocities in an individual dentinal microtubule (Text S2). The computational domain was meshed with rectangular elements and the independence of simulated results on mesh size was checked. Since the diameter of local channel (gap between terminal bead and odontoblastic process) is less than 1 µm, the influence of slip boundary (*i.e.*, non-zero flow velocity at a solid wall) on the simulated results should be considered. Therefore, the simulated results were corrected using the following equation, which has been widely accepted [37]:
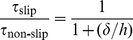
(3)where *τ*_slip_ (Pa) and *τ*_non-slip_ (Pa) are the wall shear stress when slip and non-slip boundary conditions are applied, respectively, *δ* (µm) is the slip length (the slip length is defined as the distance from the crest of the solid surface to the depth at which the linearly extrapolated velocity reaches zero at the wall (∼0.1 µm) [38], and *h* (µm) is the distance between two parallel walls (*e.g.*, local channel diameter, ∼0.12 µm).

### Modeling of nociceptor transduction

Nociceptors are the receptors for pain sensations [39], mediating the selective passage of specific ions across ion channels of a cell membrane when stimulated by noxious stimulations [40]. The passage of the ions induces an ion current through the cell membrane and generates an action potential [41]. These potentials are conducted from the peripheral sensory site to the synapse in the central nervous system and converted into neurotransmitter release at the presynaptic terminal (frequency modulation) [41]. The ion channels are generally gated by mechanical, thermal and chemical stimulations, with three different currents induced accordingly. Given the parallel distribution of ion channels in the membranes of nociceptors, the total stimulation-induced current, *I*_st_ (µA/cm^2^), may be calculated as the sum of the three:
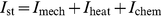
(4)where *I*_mech_, *I*_heat_ and *I*_chem_ are separately the currents generated by opening the mechanically-, thermally- and chemically-gated ion channels (all in µA/cm^2^). Since the intradental nerve terminals are stimulated by shear stress in this study, in what follows only mechanical-gated ion channels were considered for the generation of stimulation-induced current. As the gating of ion channels is a threshold process, the mechanical current (*I*_mech_) was taken as a function (*f*_m_) of the MSS (*τ*_MSS_) on the terminal bead. Consequently, *I*_mech_ can be determined by:
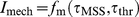
(5)where *τ*_thr_ is the mechanical pain threshold.

The relationship between the mechanical stimulation and the induced current is still unknown. However, it has been reported that the mechanical current is approximately exponentially proportional to the mechanical stimulation [27], indicating that the mechanically-gated ion channels may behave in a way similar to that of the heat-gated ion channels [28]. The quantitative relationship between the stimulation and current may thence be described as:
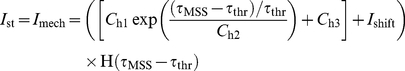
(6)where *C*_h1_, *C*_h2_ and *C*_h3_ are the constants; *H*(*x*) is the Heaviside function accounting for the threshold process; and *I*_shift_ (µA/cm^2^) is the shift current (Text S3). The constants *C*_h1_, *C*_h2_ and *C*_h3_ are set to be 2.0 µA/cm^2^, 2.0 µA/cm^2^ and −1.0 µA/cm^2^, respectively.

To the authors' best knowledge, the response kinetics of intradental nociceptors has yet been analyzed in the literature. However, all neurons have been found to behave in a quantitatively similar way as that described by the H-H model [42]. In addition, neurons exhibit various types of potassium (K^+^) conductance. The fast transient K^+^ current has been observed in a variety of neurons [43], [44]. Hence, a modified H-H model has been proposed to introduce more than one K^+^ channel to the modeling of the frequency modulation of nociceptors [45]–[47], expressed as:
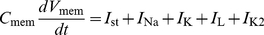
(7)


Here, *V*_mem_ is the membrane potential (mV), positive when the membrane is depolarized and negative when the membrane is hyperpolarized; 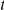 (ms) is the neural discharge time; *C*_mem_ (µF/cm^2^) is the membrane capacity per unit area; *I*_Na_, *I*_K_ and *I*_L_ are the sodium (Na^+^), K^+^ and leakage currents (µA/cm^2^), respectively; and *I*_K2_ is an additional current: the fast transient K^+^ current. *I*_Na_, *I*_K_, *I*_L_ [42] and *I*_K2_ [45] are given by:
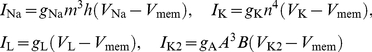
(8)where *m*, *n* and *h* are the gating variables; *A* and *B* are factors having the same functional significance as factors *m* and *h*; *V*_Na_, *V*_K_, *V*_L_ and *V*_K2_ are the reversal potentials for the Na^+^, K^+^, leakage and fast transient K^+^ currents (all in mV), respectively; and *g*_Na_, *g*_K_, *g*_L_ and *g*_A_ are the maximum ionic conductances of Na^+^, K^+^, leakage and the fast transient K^+^ currents (all in mS/cm^2^), respectively (see Text S4 for details on the determination of these variables and factors). The modified H-H model was used to model the frequency modulation of intradental nociceptors.

## Supporting Information

Figure S1 **Influence of specified LCD value** ***x*** **_1_ (at inward flow velocity of 460.4 µm/s) on simulated** ***τ*** **_thr_ and TB MSS.**
(PDF)

Figure S2 **Influence of specified LCD value** ***x*** **_1_ (at inward flow velocity of 460.4 µm/s) on simulated neural discharge rate.**
(PDF)

Text S1 **Independence of simulated neural discharge rate on specified local channel diameter value at inward flow velocity of 460.4 µm/s.**
(DOC)

Text S2 **Estimation of fluid flow velocity in an individual dentinal microtubule.**
(DOC)

Text S3 **Shift current.**
(DOC)

Text S4 **Determination of the variables and factors in the modified Hodgkin-Huxley model.**
(DOC)
