# Supplementary figures and images for: Fluid Mechanics in Dentinal Microtubules Provides Mechanistic Insights into the Difference between Hot and Cold Dental Pain
Source: PLoS One. 2011 Mar 23;6(3):e18068. doi: 10.1371/journal.pone.0018068 (PMC3063177; doi:10.1371/journal.pone.0018068)

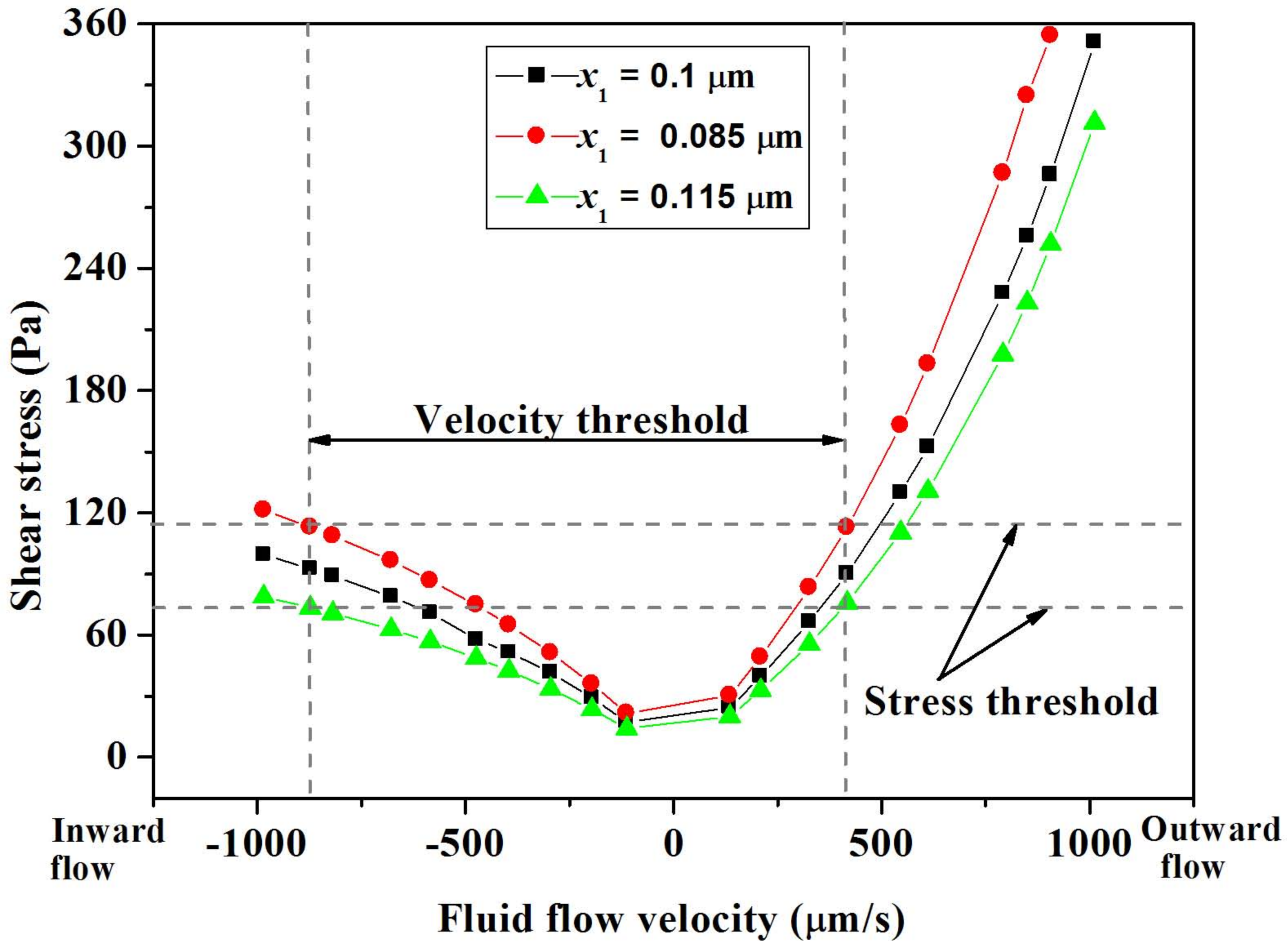

Supplement: Figure S1 — Influence of specified LCD value x 1 (at inward flow velocity of 460.4 µm/s) on simulated τ thr and TB MSS. (PDF) [file pone.0018068.s001.pdf]

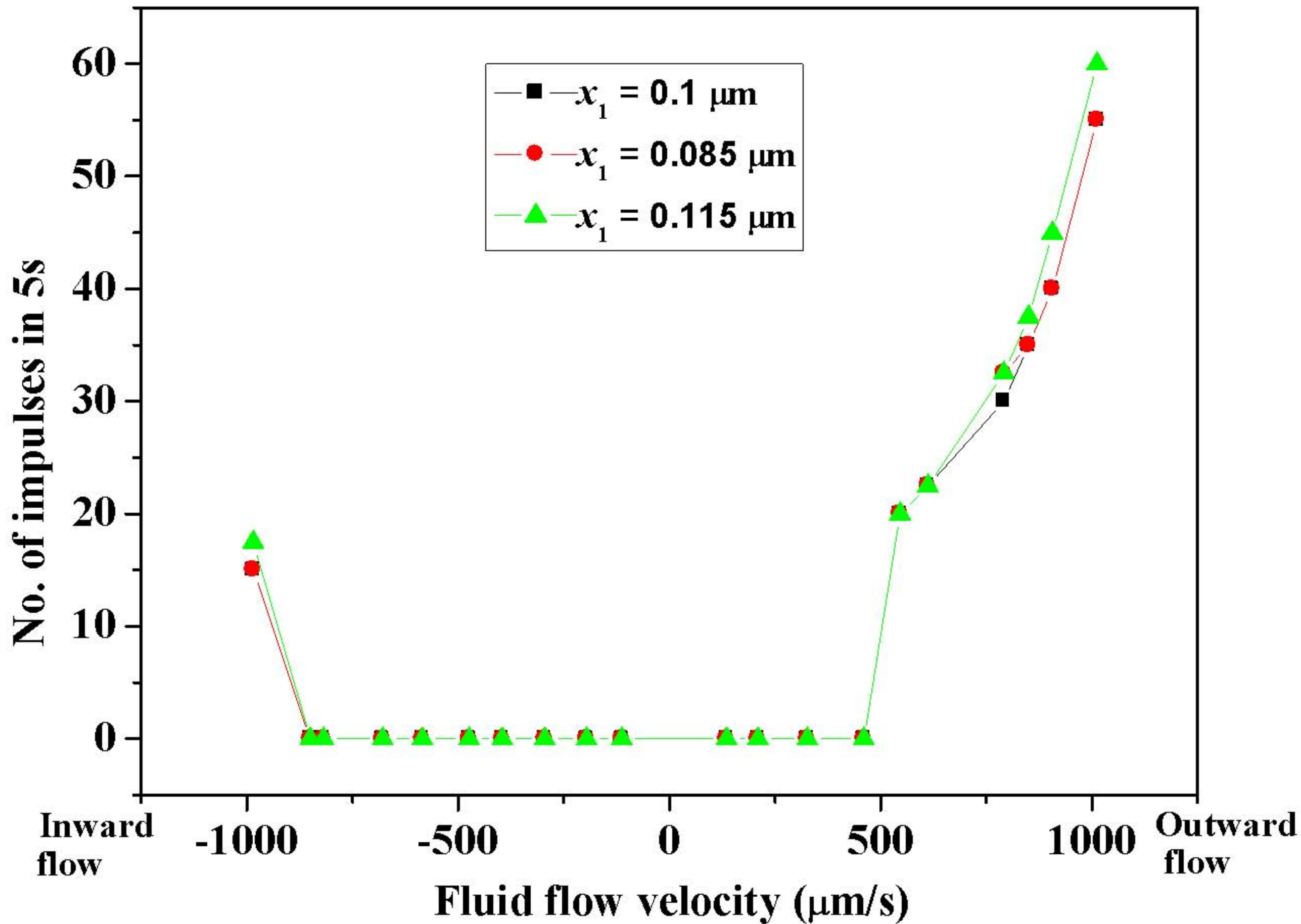

Supplement: Figure S2 — Influence of specified LCD value x 1 (at inward flow velocity of 460.4 µm/s) on simulated neural discharge rate. (PDF) [file pone.0018068.s002.pdf]
