# Supplementary material for: Fluid Mechanics in Dentinal Microtubules Provides Mechanistic Insights into the Difference between Hot and Cold Dental Pain
Source: PLoS One. 2011 Mar 23;6(3):e18068. doi: 10.1371/journal.pone.0018068 (PMC3063177; doi:10.1371/journal.pone.0018068)
Supplement: Text S1 — Independence of simulated neural discharge rate on specified local channel diameter value at inward flow velocity of 460.4 µm/s. (DOC) [file pone.0018068.s003.doc]

Independence of simulated neural discharge rate on specified local channel diameter value at inward flow velocity of 460.4 μm/s

Supplement to “Fluid Mechanics in Dentinal Microtubules Provides Mechanistic Insights into the Difference Between Hot and Cold Dental Pain”,

Lin M, Luo ZY, Bai BF, Xu F, Lu TJ

To determine the threshold shear stress (*τ*_thr_) at the inward flow velocity of 460.4 μm/s, a value (*x*_1_) is prescribed for the local channel diameter (corresponding to a channel diameter of 0.1 μm between the terminal bead and odontoblastic process). So long as *x*_1_ is prescribed, the local channel diameter values at other velocities are fixed and so does the corresponding maximum shear stress on the terminal bead (*τ*_MSS_). Fig. S1 shows that the simulated *τ*_thr_ and *τ*_MSS_ (at any flow velocity) vary with *x*_1_ (corresponding to ±15% change in local channel diameter). However, the selection of *x*_1_ appears to have little influence on the simulated neural discharge rate (see Fig. S2). This phenomenon may be explained using the stimulation-induced ion current, described as:

| $I_{\text{st}}=I_{\text{mech}}=\left(\left[C_{\text{h1}}\exp\left(\frac{\left(\tau_{\text{MSS}}-\tau_{\text{thr}}\right)/\tau_{\text{thr}}}{C_{\text{h2}}}\right)+C_{\text{h3}}\right]+I_{\text{shift}}\right)\times\text{H}\left(\tau_{\text{MSS}}-\tau_{\text{thr}}\right)$ |  |
| --- | --- |

According to the above equation, different selections of *x*_1_ result in different *τ*_thr_ and *τ*_MSS_ (both vary as *x*_1_ is varied); however, *I*_st_ hardly changes with varying *x*_1_ as it is the ratio of *τ*_MSS_ to *τ*_thr_.
