# Supplementary material for: Fluid Mechanics in Dentinal Microtubules Provides Mechanistic Insights into the Difference between Hot and Cold Dental Pain
Source: PLoS One. 2011 Mar 23;6(3):e18068. doi: 10.1371/journal.pone.0018068 (PMC3063177; doi:10.1371/journal.pone.0018068)
Supplement: Text S2 — Estimation of fluid flow velocity in an individual dentinal microtubule. (DOC) [file pone.0018068.s004.doc]

Estimation of fluid flow velocity in an individual dentinal microtubule

Supplement to “Fluid Mechanics in Dentinal Microtubules Provides Mechanistic Insights into the Difference Between Hot and Cold Dental Pain”,

Lin M, Luo ZY, Bai BF, Xu F, Lu TJ

**The average number (*N*) and mean diameter (*d*_d_) of the dentinal microtubules in cat’s canine tooth are approximately 2249/mm^2^ and 0.73 μm, respectively . Given that ~ 30% of the tubules are in free communication with the pulp , the flow velocity (μm/s) in an individual dentinal microtubule can be estimated by:**

| $V=\frac{10^{6}V}{0.3N\pi\left(\frac{d_{\text{d}}}{2}\right)^{2}}$ |  |
| --- | --- |

where *V* (nl s^-1^ mm^-2^) is the recorded flow velocity adopted from the literature .

References

1. Andrew D, Matthews B (2000) Displacement of the contents of dentinal tubules and sensory transduction in intradental nerves of the cat. J Physiol (Lond) 529: 791-802.

2. Pashley DH, Livingston MI, Reeder OW, Horner JA (1978) Effects of the degree of tubule occlusion on the permeability of human dentine, in vitro*.* Arch Oral Biol 23: 1127-1133.
