# Supplementary material for: Fluid Mechanics in Dentinal Microtubules Provides Mechanistic Insights into the Difference between Hot and Cold Dental Pain
Source: PLoS One. 2011 Mar 23;6(3):e18068. doi: 10.1371/journal.pone.0018068 (PMC3063177; doi:10.1371/journal.pone.0018068)
Supplement: Text S3 — Shift current. (DOC) [file pone.0018068.s005.doc]

Shift current

Supplement to “Fluid Mechanics in Dentinal Microtubules Provides Mechanistic Insights into the Difference Between Hot and Cold Dental Pain”,

Lin M, Luo ZY, Bai BF, Xu F, Lu TJ

According to the original Hodgkin-Huxley (H-H) model, there exists a threshold trans-membrane current for the generation of action potential. The threshold trans-membrane current varies with different selections of the relevant parameters in the model . Therefore, a shift current *I*_shift_ (μA/cm^2^) is introduced into the original H-H model to ensure that the action potential is generated when *τ*_noci_ ≥ *τ*_th_ while none is generated if *τ*_noci_ < *τ*_th_ , where *τ*_noci_ is the maximum shear stress experienced by nociceptors, *τ*_th_ is the threshold shear stress.

References

1. Hodgkin AL, Huxley AF (1952) A quantitative description of membrane current and its application to conduction and excitation in nerve*.* J Physiol 117: 500-544.

2. Xu F, Wen T, Lu TJ, Seffen KA (2008) Modeling of Nociceptor Transduction in Skin Thermal Pain Sensation*.* J Biomech Eng 130: 041013-041013.

3. Xu F, Lu TJ, Seffen KA (2008) Skin thermal pain modeling—A holistic method*.* J Therm Biol 33: 223-237.
