# Supplementary material for: Fluid Mechanics in Dentinal Microtubules Provides Mechanistic Insights into the Difference between Hot and Cold Dental Pain
Source: PLoS One. 2011 Mar 23;6(3):e18068. doi: 10.1371/journal.pone.0018068 (PMC3063177; doi:10.1371/journal.pone.0018068)
Supplement: Text S4 — Determination of the variables and factors in the modified Hodgkin-Huxley model. (DOC) [file pone.0018068.s006.doc]

Determination of the variables and factors in the modified Hodgkin-Huxley model

Supplement to “Fluid Mechanics in Dentinal Microtubules Provides Mechanistic Insights into the Difference Between Hot and Cold Dental Pain”,

Lin M, Luo ZY, Bai BF, Xu F, Lu TJ

The ion current is determined by the electrical potential difference and the conductance of membrane . The conductance of the ionic current can be regulated by the voltage dependent activation and inactivation variables (gating variables) of the conduction, given as :

| $\frac{dx}{dt}=\frac{x_{\infty}\left(V_{\text{mem}}\right)-x}{\tau_{x}\left(V_{\text{mem}}\right)}$ or $\frac{dx}{dt}=\alpha_{x}\left(1-x\right)-\beta_{x}x$ |  |
| --- | --- |

where $x$ can be any one of the three gating variables, *m*, *n* or *h*; $\tau_{x}=x_{\text{fac}}\left[1/\left(\alpha_{x}+\beta_{x}\right)\right]$ and $x_{\infty}=\alpha_{x}/\left(\alpha_{x}+\beta_{x}\right)$, *α*_x_ and *β*_x_ being the rate constants (in sec^-1^); $x_{\infty}\left(V_{\text{mem}}\right)$ is a steady-state voltage dependent (in)activation function of *x*, $\tau_{x}\left(V_{\text{mem}}\right)$ is a voltage-dependent time constant; and *x*_fac_ is a scaling factor introduced to allow greater flexibility in adjusting the firing frequency. Here, $x_{\infty}\left(V_{\text{mem}}\right)$ and $\tau_{x}\left(V_{\text{mem}}\right)$ can be calculated by :

| $x_{\infty}\left(V_{\text{mem}}\right)=1/\left(1+\exp\left[\left(V_{\text{mem}}-\vartheta_{x}\right)/\sigma_{x}\right]\right)$ |  |
| --- | --- |
| $\tau_{x}\left(V_{\text{mem}}\right)=x_{\text{fac}}\left(\tau_{x}/\cosh\left[\left(V_{\text{mem}}-\vartheta_{x}\right)/\left(2\sigma_{x}\right)\right]\right)$ |  |

where the steady-state value $x_{\infty}\left(V_{\text{mem}}\right)$ is a sigmoid function, with half of the activation (or inactivation) occurring at $V_{\text{mem}}=\vartheta_{x}$ and a slope proportional to $1/\sigma_{x}$; $\tau_{x}\left(V_{\text{mem}}\right)$ is the time constant and has a bell-shaped curve with its maximum at $V_{\text{mem}}=\vartheta_{x}$ and half-width determined by *σ*_x_; *x*_fac_ is the scaling factor. Hence, each of the gating variables is described by three parameters (*m*, *n*, *h*). From voltage clamp experiments, *α*_x_ and *β*_x_ can be approximated as :

| $\alpha_{\text{n}}=-0.01\left(V_{\text{mem}}+50\right)/\left(\exp\left[-\left(V_{\text{mem}}+50\right)/10\right]-1\right)$ |  |
| --- | --- |
| $\beta_{\text{n}}=0.125\exp\left[-\left(V_{\text{mem}}+60\right)/80\right]$ |  |
| $\alpha_{\text{m}}=-0.1\left(V_{\text{mem}}+35\right)/\left(\exp\left[-\left(V_{\text{mem}}+35\right)/10\right]-1\right)$ |  |
| $\beta_{\text{m}}=4\exp\left[-\left(V_{\text{mem}}+60\right)/18\right]$ |  |
| $\alpha_{\text{h}}=0.07\exp\left[-\left(V_{\text{mem}}+60\right)/20\right]$ |  |
| $\beta_{\text{h}}=1/\left(\exp\left[-\left(V_{\text{mem}}+30\right)/10\right]+1\right)$ |  |

The factors *A* and *B* are determined by :

| $\tau_{\text{A}}\frac{dA}{dt}+A=A_{\infty}$, $A_{\infty}=\left(0.0761\frac{\exp\left[\left(V_{\text{mem}}+94.22\right)/31.84\right]}{1+\exp\left[\left(V_{\text{mem}}+1.17\right)/28.93\right]}\right)^{1/3}$ |  |
| --- | --- |
| $\tau_{\text{A}}=A_{\text{fac}}\left(0.3632+\frac{1.158}{1+\exp\left[\left(V_{\text{mem}}+55.96\right)/20.12\right]}\right)$ |  |
| $\tau_{\text{B}}\frac{dB}{dt}+B=B_{\infty}$, $B_{\infty}=\left(\frac{1}{1+\exp\left[\left(V_{\text{mem}}+53.3\right)/14.54\right]}\right)^{4}$ |  |
| $\tau_{\text{B}}=B_{\text{fac}}\left(1.24+\frac{2.678}{1+\exp\left[\left(V_{\text{mem}}+50\right)/16.027\right]}\right)$ |  |

To the authors’ best knowledge, no data on intradental nociceptors have been reported in the literature; therefore, the parameters for a squid axon were used in the present model: *C*_mem_ = 2.8 μF/cm^2^, *g*_A_ = 47.7 mS/cm^2^, *g*_Na_ = 120 mS/cm^2^, *g*_K_ = 36 mS/cm^2^, *g*_L_ = 0.3 mS/cm^2^, *A*_fac_ = *B*_fac_ = 7.0, *m*_fac_ = *h*_fac_ = 0.263 and *n*_fac_ = 2.62. Other parameters used in the model are: *E*_Na_ = 57.19 mV, *E*_K_ = -78.78 mV , and the reversal potential of the leakage current *E*_L_ = -63.79 mV, which is obtained by adjusting *E*_L_ until the equilibrium membrane potential is achieved .

References

1. Hodgkin AL, Huxley AF (1952) A quantitative description of membrane current and its application to conduction and excitation in nerve*.* J Physiol 117: 500-544.

2. Wechselberger M, Wright CL, Bishop GA, Boulant JA (2006) Ionic channels and conductance-based models for hypothalamic neuronal thermosensitivity*.* Am J Physiol Regul Integr Comp Physiol 291: 518-529.

3. Connor JA, Walkter D, McKown R (1977) Neural repetitive firing modifications of the hodgkin-huxley axon suggested by experimental results from crustacean axons*.* Biophysic J 18: 81-102.

4. Hille B, Ionic Channels of Excitable Membranes. 1992: Sinauer Associates Inc.

5. Connor JA, Stevens CF (1971) Prediction of repetitive firing behaviour from voltage clamp data on an isolated neurone soma*.* J Physiol 213: 31-53.

6. Hodgkin AL, The conduction of the nervous impulses. 1964, Livepool: Liverpool University Press.

7. Xu F, Wen T, Lu TJ, Seffen KA (2008) Modeling of Nociceptor Transduction in Skin Thermal Pain Sensation*.* J Biomech Eng 130: 041013-041013.

8. Xu F, Lu TJ, Seffen KA (2008) Skin thermal pain modeling—A holistic method*.* J Therm Biol 33: 223-237.
